# Living in poverty and accelerated biological aging: evidence from population-representative sample of U.S. adults

**DOI:** 10.1186/s12889-024-17960-w

**Published:** 2024-02-13

**Authors:** Andrea Dalecka, Anna Bartoskova Polcrova, Hynek Pikhart, Martin Bobak, Albert J. Ksinan

**Affiliations:** 1 RECETOX, Faculty of Science, Masaryk University, Kotlarska 2, Brno, Czech Republic; 2 Department of Epidemiology and Public Health, University College London, London, United Kingdom

**Keywords:** Aging, Biological age, Biomarkers, Poverty, Socioeconomic position, Health inequalities

## Abstract

**Background:**

Biological aging reflects a decline in the functions and integrity of the human body that is closely related to chronological aging. A variety of biomarkers have been found to predict biological age. Biological age higher than chronological age (biological age acceleration) indicates an accelerated state of biological aging and a higher risk of premature morbidity and mortality. This study investigated how socioeconomic disadvantages influence biological aging.

**Methods:**

The data from the National Health and Nutrition Examination Survey (NHANES) IV, including 10 nationally representative cross-sectional surveys between 1999-2018, were utilized. The analytic sample consisted of *N* = 48,348 individuals (20-84 years). We used a total of 11 biomarkers for estimating the biological age. Our main outcome was biological age acceleration, indexed by PhenoAge acceleration (PAA) and Klemera-Doubal biological age acceleration (KDM-A). Poverty was measured as a ratio of family income to the poverty thresholds defined by the U.S. Census Bureau, adjusted annually for inflation and family size (5 categories). The PAA and KDM-A were regressed on poverty levels, age, their interaction, education, sex, race, and a data collection wave. Sample weights were used to make the estimates representative of the U.S. adult population.

**Results:**

The results showed that higher poverty was associated with accelerated biological aging (PAA: unstandardized coefficient B = 1.38 *p* <.001, KDM: B = 0.96, *p* = .026 when comparing the highest and the lowest poverty level categories), above and beyond other covariates. The association between PAA and KDM-A and age was U-shaped. Importantly, there was an interaction between poverty levels and age (*p* <.001), as the effect of poverty was most pronounced in middle-aged categories while it was modest in younger and elderly groups.

**Conclusion:**

In a nationally representative US adult population, we found that higher poverty was positively associated with the acceleration of biological age, particularly among middle-aged persons.

## Introduction

Biological aging reflects a decline in the functions and integrity of the human body and an increased vulnerability to disease [1]. Among individuals, the decline occurs with varying dynamics; therefore, people with the same chronological age may exhibit different states of their body functions [2]. Biological age higher than chronological age (biological age acceleration) indicates an accelerated state of biological aging [3] and a higher risk of premature morbidity and mortality [4].

Biological age has been employed as a convenient index for indicating the state of functional abilities. Although numerous studies assessed biological aging, there are no gold standard assays for individual biological age [5]. Numerous biological clocks have been proposed in previous studies. The most promising clocks were derived from DNA methylation and clinical biomarkers. DNA methylation-based markers of aging, also known as epigenetic clocks, represent chemical modifications of the genome that occur over the life course [6, 7]. Despite the fact that epigenetic clocks have been statistically associated with many age-related diseases and conditions (e.g., atherosclerosis, cognitive decline, menopause onset, etc.) [8, 9], such epigenetic analyses are often costly and technically challenging, making it difficult to conduct large-scale studies.

To address these limitations, analytical concepts based on clinical biomarkers that are more readily available in population studies have been developed [3, 4]. These biomarkers of aging reflect inter-individual variations in the timing of disease onset and functional decline over the lifespan [7]. To predict biological aging by clinical biomarkers, previous studies considered several statistical approaches, including multiple linear regression (MLR), principal component analysis (PCA), Klemera and Doubal’s method (KDM), as well as artificial intelligence techniques [10, 11]. For instance, Kwon and Belsky recently presented a novel R package developed to implement three methods to quantify biological aging: KDM biological age, PhenoAge and homeostatic dysregulation. The BioAge package enables users to select which biomarkers to include, then trains the algorithm using NHANES III data, which is then projected to the NHANES IV data [3].

The dynamic of aging trajectories is affected by biological factors such as sex hormone levels or genetic characteristics but, to a large extent, by modifiable health behaviors, external exposures, and socioeconomic position [4, 12]. Previous studies have suggested that individuals from socioeconomically disadvantaged backgrounds may experience accelerated biological aging [13] due to complex economic and social conditions that directly and indirectly impact health outcomes [14, 15]. Social disadvantage has been associated with increased physiological stress [16], engagement in unhealthy behaviors (e.g., inappropriate nutrition) [17], and a higher exposure to environmental risks. These exposures, accumulating across the life course, can directly influence a subsequent chain of biological processes relevant to aging [18]. Therefore, the effect of socioeconomic disadvantage on aging may vary throughout the lifespan. The results of previous studies reporting effect modification between socioeconomic status and age are mixed. Some studies found the effect of socioeconomic disadvantage more pronounced in older adults, while other studies did not find any age interaction [19]. Accordingly, to address this gap in knowledge and the need to target health and social policies to reduce health inequalities in older ages, this study aims to investigate how socioeconomic disadvantages influence biological aging in different stages of adulthood.

## Methods

### Study population

The current study used data from the National Health and Nutrition Examination Survey (NHANES) IV, an ongoing series of nationally representative cross-sectional surveys, comprising biennial surveys from 1999-2018 (10 surveys in total). Details of recruitment procedures and study design are available from the Centers for Disease Control and Prevention (Centers for Disease Control and Prevention, 2018). NHANES is a publicly available dataset (https://www.cdc.gov/nchs/nhanes) approved by the National Center for Health Statistics (NCHS) Ethics Review Board. All participants included in this study provided written informed consent.

The sample included both interviews and physical examinations, and data were collected from a variety of sources, including self-reported questionnaires, physical measurements, and laboratory tests. The baseline sample included 101,316 individuals. As the BioAge tool was calibrated on NHANES III adult individuals (age 20+), we limited the age of the sample to 20-84 years (see Methods for explanation), resulting in the sample of *N* = 54,279 individuals, from which *N* = 48,348 had available biomarker data, making this the final analytic sample. Sample weights from each survey were combined to provide nationally representative estimates for the overall sample.

### Measures

#### Biological aging mesures

The measures of biological aging used in this study were PhenoAge [7] and the Klemera-Doubal’s method [20]. These were computed using the “BioAge” R package developed by Kwon and Belsky [3, 21]. From these two measures, we derived two indices of biological age acceleration, which reflects biological age higher than individuals’ chronological age.

##### PhenoAge Acceleration (PAA)

PhenoAge is one of the most popular methods for estimating biological age. The PhenoAge measure corresponds to the chronological age at which the mortality risk would be approximately normal in a reference population. It was first described by Levine et al. by regressing the hazard of age-related specific mortality (including mortality to heart, malignant neoplasms, chronic lower respiratory disease, cerebrovascular disease, diabetes mellitus, Alzheimer´s disease, etc.) on a total of 42 clinical biomarkers and chronological age in NHANES III data. Cross-validation was further employed to select the final 9 biomarkers for the PhenoAge predictor. These 9 parameters and chronological age were then included in a parametric proportional hazards model to estimate the 10-year mortality scores that were finally converted into units of years (please see Levine et al., 2018, for a full description of the method) [7]. Kwon and Belsky provided an updated version of the PhenoAge involving 12 biomarkers [3]. We based our computation on this updated version, but we omitted C-reactive protein (CRP) as a biomarker, as this was not assessed in the 2011-2012 and 2013-2014 NHANES surveys, and used the following 11 biomarkers: albumin, alkaline phosphatase, blood urea nitrogen, creatinine, glycated hemoglobin, mean cell volume, percentage of lymphocytes, systolic blood pressure, total cholesterol, uric acid, and white blood cell count.

PhenoAge higher than chronological age indicates an advanced state of biological aging = acceleration, while PhenoAge lower than chronological age indicates delayed biological aging. Hence, PhenoAge Acceleration was calculated as the difference between PhenoAge and chronological age, where higher numbers indicated a faster biological aging.

##### KDM Acceleration (KDM-A)

For computing biological age, the Klemera-Doubal’s method (KDM) reflects an individual’s biological age at which their physiology would be considered normal (with regard to the reference sample). The KDM algorithm uses a series of regressions of chronological age regressed on selected biomarkers in a reference population, separately for males and females (please see Klemera and Doubal, 2006, for a full description of the method) [20]. KDM was found to be one of the best-performing methods in predicting mortality [21, 22]. Kwon and Belsky also provided an updated version of the KDM, with the same 12 biomarkers as for PhenoAge, and we used 11 of them.

KDM higher than chronological age reflects advanced aging while KDM lower than chronological age reflects delayed aging. Just like PhenoAge, KDM-A was calculated as the difference between KDM and chronological age, where higher numbers indicated faster biological aging.

##### Sex

Measured as male (reference group) or female.

##### Age

Coded in full years, ranging from 20 to 85 years, with individuals older than 85 years coded as 85 in the NHANES data. Because individuals older than 85 years were grouped with 85-year-old individuals, it is unknown how old these individuals were, which might bias the estimation of their biological age. For this reason, we decided to focus on individuals aged 20-84 years.

##### Education

Education level of adults (20+) coded as the highest grade of school completed, with 1 = Less than high school, 2 = High school grad/GED or equivalent, 3 = Some college or AA degree, 4 = College graduate or above.

##### Ethnicity

Measured using four categories that were available across all surveys: White, Black, Hispanic, and Other. This was recoded into three dummy codes with White as the reference group.

##### Poverty index

Measured as a total (annual) income for individuals and for other members of the family, divided by the U.S. Census Bureau poverty thresholds, adjusted for family size, and updated annually for inflation. In 2012, for instance, the average poverty thresholds were $23,492 for a family of 4 and $27,827 for a family of 5 [23]. The poverty index was assessed on a continuous scale that spanned from 1 to 5, with values extending to two decimal places. We transformed the poverty index into five distinct categories using the following criteria: 0-1 was assigned a value of 1; 1.01-2 was assigned a value of 2; 2.01-3 was assigned a value of 3; 3.01-4 was assigned a value of 4; and 4.01-5 was assigned a value of 5. Subsequently, we inverted these categories, making 1 represent the lowest level of poverty and 5 reflect the highest degree of poverty.

##### Wave

To control for possible cohort effect, the wave of data collection (coded 1-10) was used as a covariate.

### Data analysis

Our two outcome variables were PhenoAge acceleration (PAA) and KDM acceleration (KDM-A). As mentioned above, the PhenoAge and KDM indices were computed using the BioAge package. Our selected 11 biomarkers were used to train the algorithms in the NHANES III data, and the resulting estimates were then projected to the NHANES IV data. The indices of acceleration were computed by regressing participants’ estimated PhenoAge and KDM on their chronological age and exporting the residuals [24, 25]. The association between PAA or KDM-A and age was tested in a linear model with age polynomials first to see the form of the association (Model 1a for PAA and Model 1b for KDM-A). Next, the models were extended to include all the covariates (sex, age, ethnicity, education, wave of data collection, and poverty index; Model 2a for PAA and Model 2b for KDM-A). According to our research question, we also estimated interaction terms between the poverty index and age terms. R package survey was used for analyses with complex survey data design (stratified cluster-sample data with survey weights), providing nationally representative estimates. All data analyses were computed in R 4.2.1. As only a few variables (poverty ratio, PAA) had missing values with less than 10% missing, all the analyses were done with complete data.

## Results

Table 1 presents the descriptive statistics of the sample with weighted percentages. There were slightly more females in the sample (51.71%). The mean weighted age was 46.88 years, with 3.71% of participants older than 80 years. There were 68.53% White participants, 10.66% Black participants, 13.91% Hispanic participants, and 6.90% participants with different reported race/ethnicity. About 17.17% of participants reported less than high school as their highest attained education, 23.96% of participants reported completed high school, 30.95% reported attending college (without a degree), and 27.92% participants indicated college degree as their highest attained education.

**Table 1:** Descriptive statistics of the study sample

		*n*	weighted %
Sex	Male	23349	48.29
	Female	24999	51.71
Age category	20-29	8446	18.54
	30-39	8284	18.87
	40-49	8257	19.94
	50-59	7351	18.11
	60-69	7964	12.85
	70-79	5229	7.99
	80-84	2817	3.71
Race/ethnicity	White	21410	68.53
	Black	9793	10.66
	Hispanic	12670	13.91
	Other	4475	6.90
Education	Less than high school	13185	17.17
	High school grad/GED or equivalent	11278	23.96
	Some college or AA degree	13846	30.95
	College graduate or above	10521	27.92
Poverty index	Lowest poverty (1)	11432	36.31
	2	5040	13.52
	3	6811	15.50
	4	11721	20.51
	Highest poverty (5)	9153	14.16

Regarding the poverty index, 36.31% of participants were in the lowest poverty group, while 14.16% were in the highest poverty group. The descriptives of the biomarker levels are reported in Table 2.

**Table 2:** Descriptive statistics of biomarkers used in the study

	Unit	*M*	*SD*	*min*	*max*
Albumin	g/dL	4.23	0.36	2.40	5.70
Alkaline phosphatase	IU/L	71.35	23.04	7.00	210.00
Total cholesterol	mg/dL	195.62	41.61	59.00	406.00
Creatinine	mg/dL	0.62	0.13	0.10	1.51
Glycated hemoglobine (Hba1c)	%	5.66	0.88	2.00	11.30
Systolic blood pressure	mmHg	124.52	19.15	64.67	230.00
Blood urea nitrogen	mg/dL	13.45	5.31	1.00	45.00
Uric acid	mg/dL	5.41	1.45	0.40	12.50
Lymphocyte	%	30.30	8.56	2.70	73.80
Mean cell volume	fL	89.40	5.74	58.20	118.10
White blood cell count	1000 cells/uL	7.25	2.12	1.40	19.80

First, we regressed the two measures of biological aging, PAA and KDM-A, on linear and quadratic age terms (Model 1a and Model 1b). All reported estimates are unstandardized regression coefficients. The results showed a significant negative linear effect for both (PAA: B = -0.02, *p* <.001; KDM-A: B = -0.05, *p* < .001) with a positive quadratic term (PAA: B = .001, *p* < .001; KDM-A: B = .003, *p* < .001), showing that among younger NHANES participants, each additional year is actually associated with lower PAA or KDM-A. For both measures, this drop reached its lowest point at around age 50, and then the association became positive, the total association across time resembling a U shape.

In the next step, all covariates were added to these linear models. The results from the full models (Model 2a and Model 2b) are shown in Table 3. They indicated that, compared to males, females had an estimated PAA that was lower than for males (B = -2.94, *p* < .001), yet their KDM-A was higher (B = 0.74, *p* < .001). Black individuals showed higher PAA compared to White respondents (B = 0.63, *p* < .001), and a significantly higher KDM-A (B = 2.66, *p* < .001). On the other hand, Hispanic participants (B = -0.80, *p* < .001) had lower estimated PAA compared to White participants (but not KDM-A). Also, individuals of other ethnicities showed lower PAA (B = -0.55, *p* < .001) and KDM-A (B = -0.75, *p* =.048) compared to White participants. Higher levels of education were associated with lower estimated biological age for both measures, but this was only driven by college education vs less than high school (PAA B = -1.20, *p* < .001, KDM-A B = -2.43, *p* < .001), as the other levels of education were not significantly different when compared to the reference group.

**Table 3:** Results from linear models with all covariates included

	Model 2a PhenoAge Acceleration			Model 2b KDM Acceleration		
	*B*	*95% CI*	*p*	*B*	*95% CI*	*p*
Sex						
Men	(ref)			(ref)		
Women	-2.94	[-3.04, -2.84]	<.001	0.74	[0.39, 1.09]	<.001
Ethnicity/Race						
White	(ref)			(ref)		
Black	0.63	[0.47, 0.79]	<.001	2.66	[2.18, 3.14]	<.001
Hispanic	-0.80	[-0.99, -0.60]	<.001	-0.41	[-0.90, 0.08]	.098
Other	-0.55	[-0.82, -0.29]	<.001	-0.75	[-1.49, -0.01]	.048
Education						
Less than high school	(ref)			(ref)		
High school grad/GED or equivalent	0.13	[-0.02, 0.28]	.087	0.45	[-0.06, 0.96]	.082
Some college or AA degree	-0.09	[-0.25, 0.07]	.290	0.09	[-0.43, 0.62]	.721
College graduate or above	-1.20	[-1.39, -1.00]	<.001	-2.43	[-3.00, -1.87]	<.001
Wave	0.04	[0.00, 0.07]	.045	0.04	[-0.06, 0.14]	.421
Poverty index						
Lowest poverty (1)	(ref)			(ref)		
2	0.17	[-0.03, 0.36]	.094	-0.14	[-0.70, 0.42]	.621
3	0.57	[0.38, 0.76]	<.001	0.67	[0.17, 1.17]	.009
4	0.94	[0.72, 1.15]	<.001	0.91	[0.25, 1.58]	.007
Highest poverty (5)	1.38	[1.11, 1.64]	<.001	0.96	[0.12, 1.81]	.026
Age	-0.04	[-0.05, -0.03]	<.001	-0.09	[-0.11, -0.06]	<.001
Age^2^	0.002	[0.002, 0.002]	<.001	0.004	[0.002, 0.005]	<.001
Poverty*age	0.01	[0.005, 0.010]	<.001	0.02	[0.01, 0.02]	<.001
Poverty*age^2^	<-0.001	[<-0.001, <-0.001]	<.001	<-0.001	[<-0.001, <-0.001]	.017

The reference group for poverty status was the group with the lowest poverty (group 1). No effect on PAA or KDM-A was found for group 2; however, there was a significant positive effect for groups 3, 4, and 5 when compared to group 1, suggesting biological age acceleration was more pronounced in higher levels of poverty (B for the highest poverty group = 1.38 when compared to the lowest poverty group, *p* < .001 for PAA, B = 0.96, *p* = .026 for KDM-A). The linear association of age and PAA was preserved in the full model for PAA (B = -0.04, *p* < .001), as well as KDM-A (B = -0.09, *p* < .001). The quadratic association of age and PAA and KDM-A, observed in models without covariates, remained positive and statistically significant in the full model.

The relationship between poverty status and PAA was modified by age. While each unit increase in the poverty category corresponded to a 0.01 unit increase in PAA per one-year increase in age (*p* < .001), the difference among poverty index groups diminished at later age, as indicated by a significant negative interaction with quadratic age term. The same finding was observed for KDM-A, when each unit increase in the poverty categories corresponded to a 0.02 unit increase in KDM-A per one-year increase in age (*p* = < .001, and the interaction with age^2^ was also statistically significant (B < -0.001, *p* = .017).

**Fig. 1:**
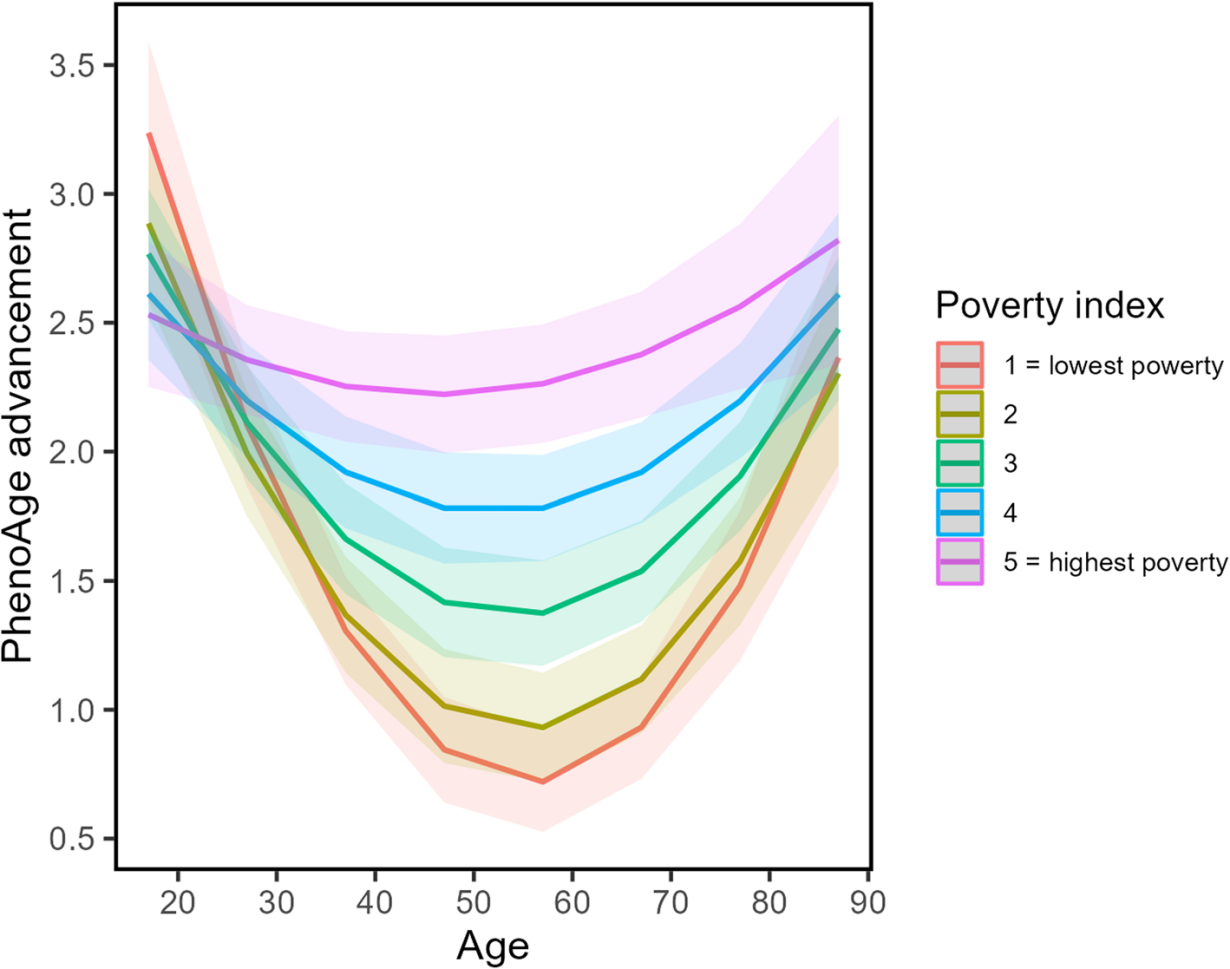
Predicted values of PhenoAge acceleration (PAA) for poverty groups across age. Note: Model controlled for sex, age, ethnicity, education, wave of data collection, and poverty index

**Fig. 2:**
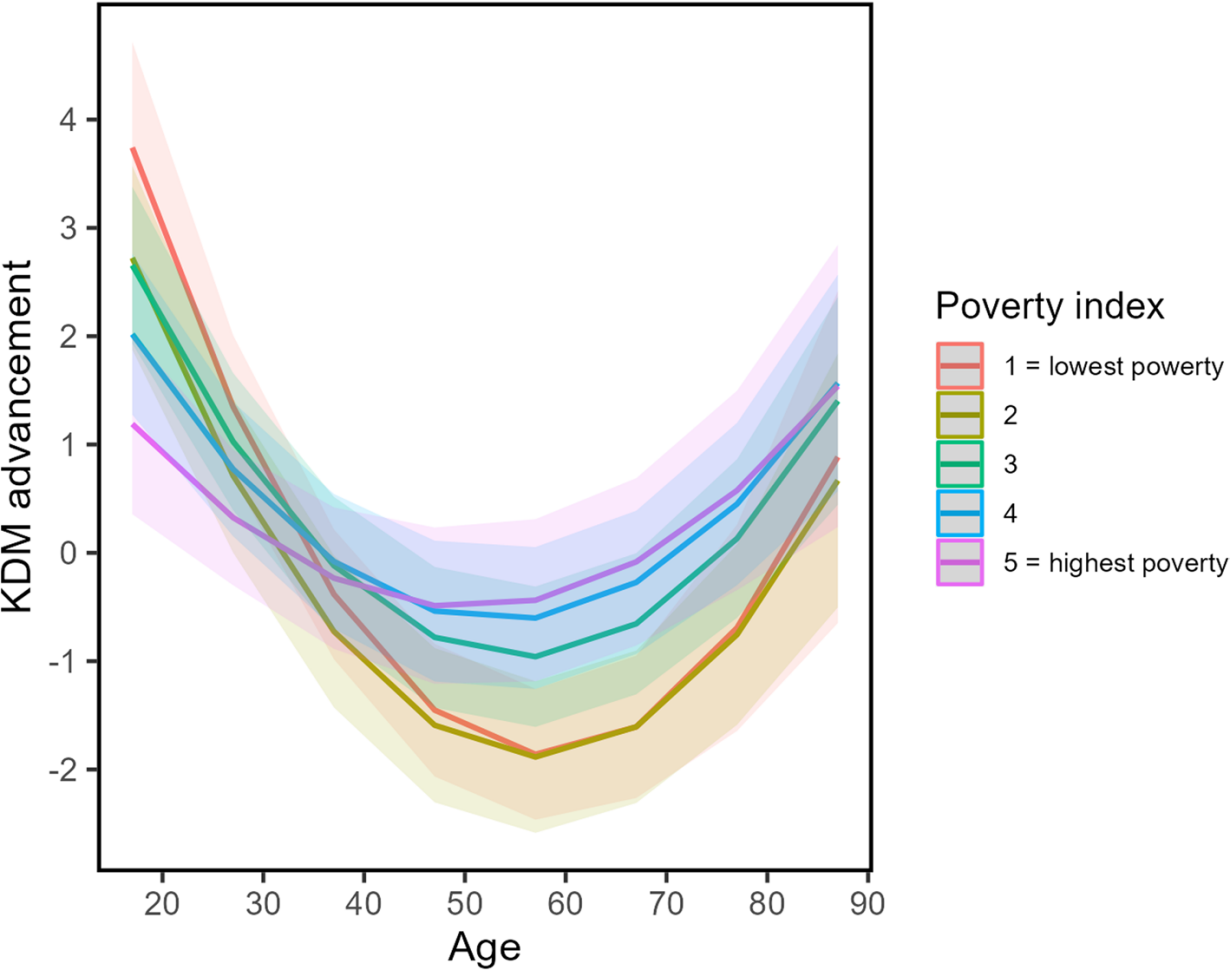
Predicted values of Klemera-Doubal’ Biological Age acceleration (KDM-A) for poverty groups across age. Note: Model controlled for sex, age, ethnicity, education, wave of data collection, and poverty index

Taken together, with increasing age, individuals with high income showed a steeper drop in PAA and KDM-A compared to individuals with lower income. Figures 1 and  2 show the predicted values of PAA and KDM-A by poverty index groups across age. Specifically, for PAA, the differences among poverty groups became most pronounced around the age of 50; afterward, the differences in PAA among poverty index groups became progressively smaller.

## Discussion

Estimating biological aging has emerged as a novel approach for understanding how socioeconomic and environmental disadvantages shape health over the life course. Our results showed that living in poverty was associated with accelerated biological aging; those in more advantageous economic positions experienced a more noticeable delay in aging, particularly in middle age, while the poverty-stricken groups showed weaker signs of this phenomenon. In the poorest group, the delay in biological aging is barely noticeable. Likewise, higher levels of education were linked with lower biological age, although a significant association was observed only in the highest educational group.

These findings extend previous observations of Crimmins et al. conducted on NHANES adults, using biological risk estimated from 9 biomarkers, including blood pressure, BMI, and blood biomarkers. The study suggested that biological risk increased more rapidly in the highest poverty group [26]. Similarly, two recent studies conducted using DNA methylation biomarkers reported significantly accelerated biological aging in adults with the lowest household incomes [27, 28].

Our results also suggested that social disparities in biological aging are substantially modified by age. In younger age groups, the socioeconomic disparities were modest but grew more pronounced with age, reaching their highest point around 50. The differences began to diminish in older age groups, becoming almost negligible in the oldest age groups. We should note that survival bias may be a possible explanation of no difference in older ages, particularly with regard to the association between poverty and life expectancy. We should also point out that the clinical biomarkers involve indicators related to chronic diseases that are commonly associated with older age. Therefore, small differences in younger ages may be explained by the generally low prevalence of risky levels of biomarkers among rich as well as poor young people. Again, our findings are consistent with the study of Crimmins et al., which reported the highest manifestation of socioeconomic disparities when individuals were in their 50s and 60s [26].

Social determinants operate at every stage of life, and no single factor is prone to drive the association between low SES and accelerated aging. Rather, adversity experienced through disadvantaged life conditions will likely have cumulative effects over the lifespan, affecting health in older age [29]. For instance, the impact of unhealthy behaviors, such as smoking, drinking, drug use, poor nutrition, and lack of physical activity may not show immediately, but such effects are chained and accumulated. For example, those who smoked, had unhealthy diets, and were physically inactive in their teens were more likely to face health issues in their 40s and 50s [30]. Similarly, low SES has been associated with living in more polluted areas that may, in the long-term, promote a faster decline of functional abilities of the organism (e.g., lung functions, cognitive functions, etc.) [31, 32]. Finally, low wealth has been related to greater chronic life stress that may influence mental well-being [33].

### Strength and limitations

This study involved a large population-representative sample of U.S. adults enrolled across two decades (1999-2018). We measured biological age using two measures that are well-validated as predictors of age-related conditions and diseases based on previous studies [34, 35]. PAA and KDM-A were quantified using readily available clinical biomarkers. We selected the poverty income ratio as a more precise indicator of contemporary socioeconomic resources than measures such as education and occupational status. The ratio better addresses the economic stability of the individuals, family income, and spatial and temporal differences in financial needs [36].

Several limitations need to be acknowledged. First, our analyses were conducted using cross-sectional data without repeated measurements of biological aging biomarkers and predictors. Thus, we were unable to observe temporal relationships between early-life exposures to socioeconomic disadvantages and biological aging in late adulthood. Also, it limits our ability to study important mediation effects of unhealthy behaviors (e.g., smoking, alcohol consumption, unhealthy diet, etc.) that may occur as a consequence of living in a socioeconomically disadvantaged environment, and that may subsequently accelerate biological aging later in life. Second, we should note that our analyses depended on PAA and KDM-A that were estimated based on blood biomarkers only and did not include several essential biomarkers of functional abilities, including cognitive performance, pulmonary functions, or physical fitness, which are suitable predictors of healthy aging [37]. In addition, epigenetic clock information, an increasingly popular approach to estimate biological aging, was not available in the data. DNA methylation-based markers have shown impressive accuracy with chronological age [38] and have been associated with many age-related diseases and conditions [8]. Third, we had no information about the health status of the participants, which might potentially affect the representativeness of the study sample, as individuals with poor self-reported health are less likely to participate in the study. However, we partially addressed this issue by utilizing sample weights in all data analyses.

## Conclusion

The world population is experiencing an unprecedented increase in the percentage of older people, and biomarkers of aging may empower the evaluation of interventions for promoting healthier aging [39]. Further, it is essential to determine whether the additional years of life will be spent mainly in good or poor health. Evaluating the biological age over the lifespan is a simple but convenient tool for indicating the state of functional abilities. Our study suggests that economic disadvantage is associated with accelerated aging. Additionally, we identified an age window of the highest manifestation of social disparities in health. Thus, our results highlight the importance of a life course perspective in research as well as the need to tackle the burden of health inequalities to enable healthy aging for all.

## Data Availability

NHANES is a publicly available dataset (https://www.cdc.gov/nchs/nhanes).
